# Point-spread function reconstructed PET images of sub-centimeter lesions are not quantitative

**DOI:** 10.1186/s40658-016-0169-9

**Published:** 2017-01-13

**Authors:** O. L. Munk, L. P. Tolbod, S. B. Hansen, T. V. Bogsrud

**Affiliations:** 1 Department of Nuclear Medicine & PET Centre, Aarhus University Hospital, Aarhus, Denmark; 2 Department of Radiology and Nuclear Medicine, Oslo University Hospital, Oslo, Norway

**Keywords:** Positron emission tomography, Reconstruction, Point spread function, Standardized uptake value (SUV), Quantitative imaging, Image artifacts

## Abstract

**Background:**

PET image reconstruction methods include modeling of resolution degrading phenomena, often referred to as point-spread function (PSF) reconstruction. The aim of this study was to develop a clinically relevant phantom and characterize the reproducibility and accuracy of high-resolution PSF reconstructed images of small lesions, which is a prerequisite for using PET in the prediction and evaluation of responses to treatment.

Sets of small homogeneous ^18^F-spheres (range 3–12 mm diameter, relevant for small lesions and lymph nodes) were suspended and covered by a ^11^C-silicone, which provided a scattering medium and a varying sphere-to-background ratio. Repeated measurements were made on PET/CT scanners from two vendors using a wide range of reconstruction parameters. Recovery coefficients (RCs) were measured for clinically used volume-of-interest definitions.

**Results:**

For non-PSF images, RCs were reproducible and fell monotonically as the sphere diameter decreased, which is the expected behavior. PSF images converged slower and had artifacts: RCs did not fall monotonically as sphere diameters decreased but had a maximum RC for sphere sizes around 8 mm, RCs could be greater than 1, and RCs were less reproducible. To some degree, post-reconstruction filters could suppress PSF artifacts.

**Conclusions:**

High-resolution PSF images of small lesions showed artifacts that could lead to serious misinterpretations when used for monitoring treatment response. Thus, it could be safer to use non-PSF reconstruction for quantitative purposes unless PSF reconstruction parameters are optimized for the specific task.

**Electronic supplementary material:**

The online version of this article (doi:10.1186/s40658-016-0169-9) contains supplementary material, which is available to authorized users.

## Background

The spatial resolution and signal-to-noise ratio of PET images have improved significantly due to image reconstruction methods that include modeling of resolution degrading phenomena, particularly detector effects such as crystal size, inter-crystal scattering, and crystal penetration, but also positron range and angle deviation from 180° during electron-positron annihilation. This more accurate system matrix is used in statistical reconstruction algorithms, also referred to as resolution recovery, resolution modeling, or point-spread function (PSF) reconstruction [1, 2]. Today, all major vendors of clinical whole-body PET/CT systems provide PSF reconstruction algorithms for clinical PET imaging. PSF reconstruction produces images with improved isotropic spatial resolution, reduced spill-in/spill-out, and ultimately increased activity concentration (Bq/mL) or standardized uptake value (SUV) in small lesions that are thus more easily detected and characterized. These benefits have been demonstrated as higher recovery coefficients (RCs) in NEMA phantom studies [3] and improved lesion detectability in patient studies [4].

There is a growing interest for using PET in the prediction and evaluation of early responses to treatment, such as chemotherapy, radiotherapy, and local ablative therapy, e.g., of liver metastases, in order to identify non-responders as soon as possible to optimize their treatment strategy [5–8]. PET images used to plan and evaluate therapeutic strategies should have a high spatial resolution and good contrast-to-noise ratio, but foremost the measurements need to be *quantitative* and *reproducible*. PSF images have distinct noise texture [3, 9] and artifacts such as edge overshoot and hyper-resolution of focal uptake [10–12]. These unwanted effects on the quantification of small structures need to be thoroughly studied in a clinical relevant setting as these artifacts are not revealed by the standard NEMA image quality test [13]. The NEMA image quality measurement is widely used to compare the image quality of different imaging systems where a phantom containing six fillable spheres of different sizes (10–37 mm diameter) are imaged so that the hot spheres have a fixed activity concentration four and eight times that of the background. However, NEMA phantom and other commercially available phantoms have limitations with respect to evaluation of small lesions as their spheres are too large compared to the size of small lesions that can be detected in clinical practice. Furthermore, the fillable acrylic glass spheres in the phantoms separate the hot spheres from the background activity by a non-radioactive layer, which does not mimic the physiologic reality and cause quantitative errors [14], particularly for small diameters.

In this study, we developed a new phantom to produce clinically relevant measurements to study the performance of PSF reconstruction algorithms for the quantification of small sub-centimeter lesions in direct contact with surrounding tissue. We scanned phantoms with small sub-centimeter spheres at a wide range of sphere-to-background ratios (1:1 to 15:1). PET data were acquired on PET/CT systems from two vendors, we reconstructed images with and without PSF, and we explored effects of convergence and post-reconstruction filtering. We measured activity concentrations and RCs based on volumes of interests (VOIs) commonly used for cancer diagnostics to assess whether PSF reconstructed images of small lesions provide accurate quantification, which is needed for treatment response monitoring or tracer kinetic modeling.

## Methods

### Preparation of wall-less sub-centimeter sphere phantoms

A 50-mL solution of ^18^FDG was mixed with 4.5-g gel powder (Orthoprint, Zhermack Clinical, Italy) by rigorously shaking for 30 s. The homogeneous mixture was drawn into a syringe and injected into a row of 12 spherical molds sitting along a thin 30-cm string. The gel was allowed to settle for around 2 min before the 12 hot spheres (diameters in mm: 10, 10, 6, 6, 12, 4, 10, 3, 8, 4, 4, and 6) were released (see Additional file 1). Three sets of hot spheres were made from the same ^18^FDG-solution and carefully inspected for imperfections such as air bubbles. The first set was suspended in free air. The second set was suspended and covered by 250-mL two-component silicone (Magic Gel 1000, Raytech, Italy) that provided a scattering medium. The third set was embedded in 250-mL ^11^C-silicone that was prepared by mixing a small volume of ^11^C solution into one of the silicone components using a magnetic stirrer before adding the second component, which allowed us to study the spheres at varying sphere-to-background ratios. We verified that ^18^FDG did not move by diffusion from the spheres into the silicone. New phantoms were prepared for each PET scan and placed in the scanner with a large source of the ^18^FDG-reference (around 25 mL remaining from the sphere production). The sphere production method using molds was originally developed by Skretting and co-workers [15], and in this study it was further improved by the surrounding non-diffusible scatter medium that allowed us to construct a clinical relevant phantom mimicking small homogeneous hot lesions in direct contact with surrounding tissue.

### Phantom scans

PET list mode data acquisitions were performed on two widely used clinical PET/CT systems: Siemens Biograph 64 Truepoint TrueV (Siemens Healthcare, Erlangen, Germany) and GE Discovery 690 (GE Healthcare, Milwaukee, WI, USA). The scanners have similar spatial resolutions when measured according to the NEMA NU2-2007 using filtered back projection [13]: 4.4 [16] and 4.7 mm FWHM [17]. Both PET/CT systems include iterative PSF reconstruction algorithms that further improve the spatial resolutions. List mode data were divided into 10 min frames and reconstructed with PSF. The data were decay-corrected using the ^18^F half-life and corrected for scatter and attenuation using CT images. For Siemens Biograph 64, list mode data were separated in prompt and random sinograms, and images were reconstructed using attenuation weighted ordered subset expectation maximization without PSF (Iterative3D) and with PSF (TrueX). For GE Discovery 690, list mode data were reconstructed using VuePoint FX SharpIR algorithm with time-of-flight (TOF) and PSF. Iterative reconstruction algorithms without resolution modeling are not available for GE Discovery 690. We explored the reconstruction algorithms with varying number of iterations (keeping the number of subsets constant), width of post-filters, and size of the reconstruction matrices with focus on high-resolution imaging of small lesions (see figures for details).

### Image analyses

Each PET image of the phantoms was analyzed as follows: the true activity concentration in the spheres was measured using a 6-mL cube-shaped VOI placed on the large homogenous ^18^FDG-reference, and the background activity concentration was measured using a cube-shaped 15-mL VOI placed on the ^11^C-silicone. The large VOIs were not expected to be affected by PSF artifacts and partial volume effects [18]. For the spheres suspended in ^11^C-silicone, the true sphere-to-background ratio was calculated as true activity concentration in the spheres divided by background activity concentration, representing the average activity ratio during the 10-min time frames. In the last PET frame, a centrally placed sphere-shaped VOI was defined on each sphere with the same diameter as the physical sphere. Then, frame-by-frame, the sphere-shaped VOIs were used to enclose three commonly used metrics: maximum activity concentration (A_max_), background-corrected 3D isocontour at 50% max (A_50bg_), and average activity concentration (A_avg_). A_max_ and A_50bg_ are measures that are available on most visualization tools and therefore reflect how physicians perceive lesions during image reading when extracting SUV measures [19]. In contrast, A_avg_ is rarely used clinically because it requires a VOI that exactly covers the lesion, and the exact dimensions of a lesion are usually not known and cannot be reliably deduced from clinical PET images (particularly not for the smallest diameters). The activity concentrations were used to calculate RCs (RC_max_, RC_50bg_, and RC_avg_) defined as the measured activity concentration divided by the true activity concentration. The RCs and the true sphere-to-background ratio were calculated frame-by-frame in the dynamic series. Both measures are ratios and independent of decay correction. The shorter half-life of the ^11^C background generated an increasing true sphere-to-background ratio as function of time.

## Results

### Visual inspection of hot spheres in free air

**Fig. 1:**
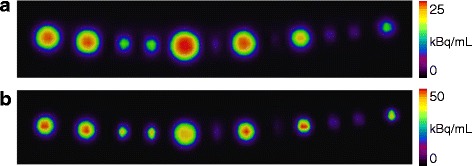
The spheres in air (diameters in mm: 10, 10, 6, 6, 12, 4, 10, 3, 8, 4, 4, and 6) scanned on a Siemens Biograph 64 Truepoint PET/CT. Two reconstructions of the same PET data are shown. **a** Without PSF (Iterative3D, 6 iterations, 21 subsets, 2-mm Gaussian filter, 336 × 336 matrix) and **b** With PSF (TrueX using same reconstruction parameters including PSF). Note that different intensity scales were used on the two images to allow visualization of all spheres (RCs are higher with PSF). Without PSF, the well-known pattern is seen: the RCs drop monotonically as function of sphere diameter. With PSF, the rank order of RCs is: 8, 10, 12, 6, 4, and 3 mm

PET data of a set of hot spheres suspended in free air were reconstructed with and without PSF (Fig. 1). Visual inspection of the images reconstructed without PSF (Fig. 1a) revealed the expected monotonic relation between the measured activity concentration and the size of a sphere [18]. All spheres contained the same activity concentration, but limited spatial resolution and spill-out lead to monotonically lower RC (lower measured activity concentration) for smaller spheres. On the PSF image (Fig. 1b), the spheres had higher measured activity concentration, but the relation between the measured activity concentration and the size of a sphere was non-monotonic with a maximum value around 8 mm. The experiment was reproduced with a new set of spheres suspended in free air that was scanned on three different Siemens Biograph TrueV PET/CT systems with the same results: a monotonic relation between RC and sphere size on images reconstructed without PSF, and a non-monotonic relation with a maximum value at 8 mm on the PSF images.

### Recovery as function of sphere size and sphere-to-background ratio

**Fig. 2:**
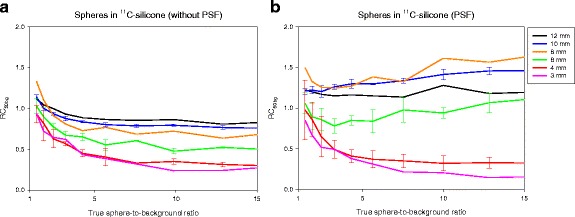
Spheres in ^11^C-silicone scanned on Siemens Biograph 64 Truepoint PET/CT. Two image series based on the same PET raw data were analyzed. **a** Without PSF (Iterative3D, 4 iterations, 16 subsets, 2-mm Gaussian filter, 336 × 336 matrix, voxel size 2.0 × 2.0 × 2.0 mm^3^); and **b** With PSF (TrueX with PSF, 4 iterations, 21 subsets, 2-mm Gaussian filter, 336 × 336 matrix, voxel size 2.0 × 2.0 × 2.0 mm^3^). The x-axes are the true sphere-to-background ratio from 1:1 to 15:1. The y-axes are RCs measured as RC_50bg_. For 10, 6, and 4 mm spheres, the lines are mean values and error bars are ± standard deviation. With PSF, the RC_50bg_ can be > 1, and in general the 8–10 mm spheres have the highest RCs

**Fig. 3:**
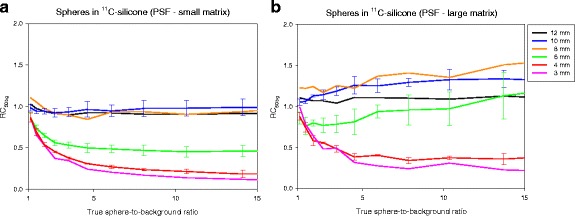
Spheres in ^11^C-silicone scanned on GE Discovery 690 PET/CT. Two image series, both with PSF, based on the same PET raw data were analyzed. **a** VuePoint FX SharpIR with TOF and PSF (3 iterations, 24 subsets 4-mm 2D Gaussian filter, and [1, 1, 6] axial convolution filter, 192 × 192 matrix, voxel size 3.7 × 3.7 × 3.3 mm^3^) with moderate spatial resolution; and **b** VuePoint FX SharpIR with TOF and PSF (4 iterations, no post-filter, 256 × 256 matrix, voxel size 2.0 × 2.0 × 3.3 mm^3^) with higher spatial resolution. VOI definition, symbols, and axes are as described in Fig. 2. Similar to Fig. 2b, this PSF algorithm also leads to images where RCs can be non-monotonic function of sphere size with RC_50bg_ > 1, and with the highest RCs for 8–10 mm spheres

Three sets of spheres were prepared from the same ^18^FDG-solution and suspended in free air, silicone and ^11^C-silicone. The three sets were placed in the scanner with an ^18^FDG-reference, and list mode data were acquired. Images were reconstructed with and without PSF. Figure 2 shows the RC_50bg_ as function of the true sphere-to-background ratio for the spheres suspended in ^11^C-silicone that were scanned using Siemens Biograph Truepoint TrueV PET/CT. All RCs were initially around 1, since the initial sphere-to-background was around 1 resulting in equal amount of spill-in and spill-out of signal between spheres and the background [18]. For the images reconstructed without PSF (Fig. 2a), the monotonic relation between RC and sphere size developed as the true sphere-to-background ratio increased, and spill-out from spheres to the background became more important. For the PSF images (Fig. 2b), the RCs were slightly increasing for the 6–10 mm spheres as the true sphere-to-background ratios increased, whereas the RCs for the smallest spheres (3–4 mm) decreased as the true sphere-to-background ratios increased. The non-monotonic relation between RC and sphere size was independent of the true sphere-to-background ratio. The experiment was repeated on a GE Discovery 690 PET/CT scanner with a new set of spheres. Figure 3 shows two PSF reconstructions from the GE Discovery 690 PET/CT where the higher spatial resolution on Fig. 3b (large matrix) leads to higher RCs, particularly for the 6-mm sphere, and exactly the same distinct features and non-monotonic relation between RC and sphere size as PSF images from the Siemens Biograph Truepoint TrueV PET/CT. RC_max_ curves were similar RC_50bg_ but noisier (data not shown).

### Reproducibility of RC measurements

**Fig. 4:**
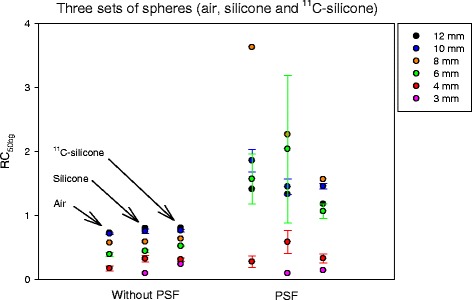
The three sets of spheres made from the same ^18^FDG-solution were scanned using Siemens Biograph 64 Truepoint PET/CT. PET raw data from the late part of the dynamic scan, for a true sphere-to-background ratio around 13:1, were reconstructed using without and with PSF (same reconstructions as in Fig. 2). For each sphere size, RCs should be similar as seen for reconstruction without PSF. However, RCs were less reproducible and non-monotonic when using PSF reconstruction. For 10, 6, and 4 mm spheres, the symbols are mean values and error bars are ± standard deviation (*N* = 3). The 3-mm sphere in free air was missing.

For images without PSF, the RCs followed the same and reproducible pattern for each sphere size, which depends on the surrounding medium: RC_air_ ≤ RC_silicone_ ≤ RC_11C-silicone_ (Fig. 4). This also showed that the phantom production was highly reproducible. In contrast, PSF images reconstructed using the same PET raw data lead to higher, non-monotonic, and less reproducible RCs.

### RC as function of iterations

**Fig. 5:**
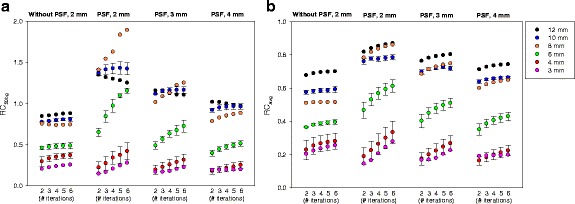
Spheres in ^11^C-silicone scanned on Siemens Biograph 64 Truepoint PET/CT. RC_50bg_ (**a**) and RC_avg_ (**b**) as function of iterations and sphere size for true sphere-to-background ratio around 8:1. Images were reconstructed with varying iterations (the number of subset was kept constant: 16 without PSF and 21 with PSF) and post-filter widths using a 336 × 336 × 109 matrix with a voxel size 2.0 × 2.0 × 2.0 mm^3^. For 10, 6, and 4 mm spheres, the symbols are mean values and error bars are ± standard deviation. Note: the VOI used for the 3 mm consists of only 1 voxel. Thus, for the 3-mm sphere RC_avg_ is equal to RC_max_. With PSF and a narrow (2 mm) post-filter, the RC measures are non-monotonic function of sphere size (maximum 8–10 mm diameter); whereas wider post-filters suppress the PSF artifacts. Note the different scales on the y-axes

The three metrics (A_max_, A_50bg_, and A_avg_) all exhibited the same trend where RCs increased with increased number of iterations. The RCs for both maximum-based metrics were similar but with RC_max_ (data not shown) being slightly higher than RC_50bg_. The convergence speed (RCs vs iterations) was markedly slower when using PSF reconstruction (Fig. 5). For the smallest 3–4 mm spheres, PSF reconstruction could in fact lead to lower RCs than reconstruction without PSF, but RCs were always found to increase with number of iterations. Without PSF, RC_50bg_ was a monotonically increasing function of sphere size and always less than 1. With PSF, a non-monotonic relation between RC and sphere size was pronounced when using many iterations and narrow post-filters. The 8-mm sphere had a particularly high RC_50bg_. RC_avg_, which is based on a large VOI with volume equal to the physical sphere, was more robust to edge overshoot caused by PSF reconstruction than maximum-based measures such as RC_50bg_ (Fig. 5b).

### Optimized reconstruction parameters

The majority of the tested combinations of iterations and post-filter widths lead to RC_50bg_ larger than 1 and a clearly non-monotonic relationship between RC and sphere size (Fig. 5a). However, the use of wider post-filters suppressed the PSF artifact and restored the monotonic relationship but at the cost of lower spatial resolution. Very wide post-filters removed the PSF artifacts but also the benefit of higher RCs using PSF reconstruction. For the data in Fig. 5a, there was not one optimal set of reconstruction parameters for all sphere sizes. The use of 4 iterations, 21 subsets, and a 3-mm Gaussian filter produced images that restored the monotonic relation between RC and lesion size while still providing higher RCs than images reconstruction without PSF for sphere sizes around 6–12 mm. The PSF artifacts were much less pronounced when images are evaluated using A_avg_ (Fig. 5b). RC_avg_ values were always less than 1, and only for the narrow 2-mm post-filter a non-monotonic behavior was observed for all iterations.

## Discussion

Contemporary reconstruction algorithms all include PSF, which provide improved spatial resolution and better lesion detectability [4]. In this project, we developed a phantom to mimic small lesions in a patient, which allowed us to evaluate the accuracy and precision of RCs in small lesions. Our results confirm previous findings that PSF reconstruction leads to higher lesion detectability. However, we found that PSF reconstruction produced image artifacts such as RCs that are not a monotonic function of lesion size (Fig. 1) for all tumor-to-background ratios (Figs. 2 and 3), and RCs that are noisy and could have values greater than 1 (Fig. 4). It is crucial to understand the PSF artifacts otherwise they can lead to serious unwanted consequences when using SUV measures to monitor disease and treatment response. We note that the PSF artifacts are present on two different PET/CT systems (Figs. 2 and 3) and are thus not caused by vendor-specific implementations of PSF in their reconstruction algorithms.

A limitation of this study is that our phantom did not include all challenging characteristics of real patient scans such as varying patient sizes, image noise, location and shape of lesions, and respiratory movement, but the setup was useful to optimize PSF reconstruction parameters for quantification. The experiment including production of the spheres was highly reproducible as seen for reconstruction without PSF (Fig. 4). For the three sets of spheres, the RCs depended on the surrounding medium as expected. In free air, the positron range is large leading to the lowest RCs, with silicone as scattering medium the RCs will be slightly higher, and with ^11^C-silicone background there will be less spill-over and RCs will be highest. Thus, for a late image in the dynamic series with low ^11^C-background activity, similar RCs should be expected for spheres of the same size but with a tendency: RC_air_ ≤ RC_silicone_ ≤ RC_11C-silicone_. However, RCs were less reproducible for PSF reconstruction.

**Fig. 6:**
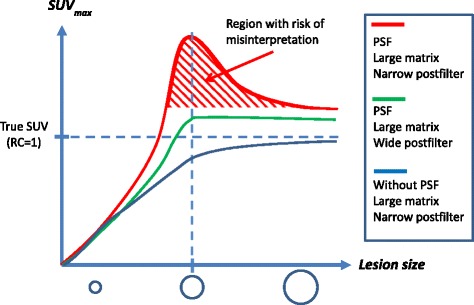
Schematic illustration regarding the challenges in choosing optimal reconstruction parameters in a clinical setting. The SUV_max_ values are shown as a function of lesion size (SUV curve) up to 12 mm for lesions with identical uptake of tracer, e.g., ^18^FDG. *Red*: highest spatial resolution but with risk of misinterpretations due non-monotonic SUV curve (probably best for lesion detection). *Green*: a slightly wider post-filter restored the expected monotonic SUV curve but at the cost of decreased detectability (probably best for response evaluation). *Blue*: without PSF (not recommended for lesion detection. Useful for response evaluation but inferior to optimized PSF reconstruction, such as the green curve). The SUV curve shapes in the figure were based on recovery measures from Fig. 5. SUV curves are scanner-specific and depend on reconstruction algorithm, reconstruction parameters, and PSF kernel size. Note that *SUV curves* are only non-monotonic for high-resolution imaging of sub-cm lesions, such as normal sized lymph nodes and small lung lesions

In practice, the physical size of a lesion is difficult to derive from PET images due to spill-out and partial volume effects [18]. Thus, in clinical studies the quantification is generally based on the maximum voxel value or the average values inside a 3D isocontour [19], and we have shown that this may lead to wrong conclusions. As an example, imagine a patient with a small (sphere-shaped) tumor being treated with 6 cycles of chemotherapy. After 2 cycles, the tumor diameter shrinks from 12 to 8 mm with the remaining tumor tissue having unchanged physiology. The treatment appears to be effective, the tumor volume has been reduced by more than a factor 3, and the patient has a chance to be completely cured after the remaining treatments. However, the patient had a PET scan acquired after 2 cycles of chemotherapy being compared to a pretreatment PET baseline scan. As illustrated in Fig. 1b (compare the 12-mm sphere to the 8-mm sphere), the conclusion based on SUVs could be that SUV_max_ has increased after the treatment, which usually indicates disease progression. This misinterpretation is caused by PSF artifacts and could lead to a wrong decision, e.g., to change or stop an effective treatment. Thus, it is important to optimize image reconstruction parameters to suppress PSF artifacts for images used for treatment response monitoring because the artificial non-monotonic relation between SUV and lesion size is counterintuitive based on previous experiences from the PET literature as illustrated in Fig. 6. We expect that this PSF artifact is only present for high-resolution imaging of small lesions, as studied here, whereas the RC for larger lesion should not be affected by edge overshoot using PSF reconstruction. For larger lesions, average-based RCs should approach 1 with maximum-based RCs being a bit higher due to noise.

Our results indicate that for the detection of the smallest lesions (3–4 mm diameter) PSF reconstruction can perform worse than reconstruction without PSF, when using few iterations as commonly recommended by the vendors. PSF reconstruction always improves detection for the larger lesions (6–12 mm diameters). These results may be explained by lack of convergence, and similar observations have been made in studies of small lesions and myocardial defects [20, 21] that also found that TOF reconstruction require fewer iterations to converge. Figure 5 illustrates that inclusion of PSF delays convergence. Without PSF, RCs were monotonic functions of sphere size and slightly increased as function of iterations. With PSF, the maximum-based measures, RC_50bg_ and RC_max_, converged slower than RC_avg_ (compare Fig. 5a–b) probably because the maximum voxel value is more dependent on higher-frequency features, voxel noise and edge artifacts in the image. For all spheres, RC_avg_ and RC_max_ continued to increase as function of iterations (we stopped at 6 iterations 21 subsets, which is more than commonly used). This effect was particularly pronounced for the 6–8 mm spheres when using the 2-mm post-filter. The overshooting artifact leading to RC > 1 could be related to a broad PSF kernel. It has been shown that narrower PSF kernel reduces the overshooting artifact [2, 22], and that the artifact persists even when the width of the PSF matches the size of the system’s PSF [23]. It is not possible to change the PSF kernel width on commercial clinical scanners, but our results indicate that their standard PSF kernels may be too broad. However, wider post-filters suppressed the PSF artifacts and restored the expected relation that the RC fall monotonically as the sphere size decreases (Fig. 5), but the post-filter should not be too wide because this would unnecessarily reduce the RCs. As an example, TrueX with 4 iterations, 21 subsets, and a 3-mm Gaussian filter produced images that suppressed edge artifacts sufficiently to restore the expected monotonic relation between RCs and sphere size, but still provided maximum-based RCs that can be slightly larger than 1 (e.g., up to 1.2 for RC_50bg_). The wider 4-mm Gaussian filter was needed to also avoid RCs larger than 1, while still providing RCs that are around 20% higher than without PSF for spheres larger than 6 mm diameter.

The best choice of PSF reconstruction parameters will be a trade-off between lesion detectability and avoiding PSF artifacts affecting quantification, which should be optimized for the specific task. In this work, we studied accuracy and precision of RCs (and SUV values) that allowed us to optimize PSF reconstruction parameters for quantification. For detection, the PSF reconstruction parameters should optimize the signal-to-background ratio while also accounting for image noise, which was not studied here. PSF reconstruction leads to decreased noise at the individual voxel level but with increased correlation among nearby voxels, which leads to distinct noise texture being less granular but lumpier [9, 22]. The lumpy noise decreases reproducibility when quantifying VOIs of a similar or smaller size as the PSF noise structures, as we have demonstrated (Fig. 4). Thus, PSF images should be used with caution for quantitative analyses of small lesions.

The phenomena described are relevant not only for the measurement of SUV but for visual assessment of PET images, as well. In this work, we have studied the performance of PSF reconstruction algorithms for the quantification of tracer uptake in small, sub-centimeter lesions at varying lesion-to-background ratios. It must be anticipated that the phenomena described is valid for thin tissue layers, as well, e.g., pleura and peritoneum, the narrow periphery of cystic and necrotic tumors and for vessel walls. Also, variation in apparent cortical brain uptake could be caused by variation in cortical thickness rather than variation in metabolic activity. As a result of the same phenomena, it must also be anticipated that the SUV might be biased in small liver lesions (e.g., liver cysts) with reduced uptake compared to surrounding normal liver tissue, as well as in the lumen of arteries with increased uptake in the vessel wall (e.g., arteritis). In these special cases, the quantitative properties of high-resolution PET images should be validated using dedicated phantoms preferably avoiding artificial non-radioactive layers caused by plastic walls in commercial fillable phantoms [14]. A recent study explored the use of PSF reconstructed images for volume segmentation in delineation of tumor volumes in radiotherapy planning [24]. Compared to our study, they used a NEMA phantom with extra spheres and reconstructed images using wider filters, i.e., with lower spatial resolution, but interestingly, they identified problems related to larger spheres and recommended not to use PSF reconstruction for volume segmentation using adaptive thresholding methods. For multicenter studies, the measured SUV values are further impacted by the use of different scanners and reconstruction algorithms. NEMA studies can be made to harmonize SUV quantification, and a method to make SUV values comparable without applying wide post-filters that would obscure lesion detection has recently been suggested [25]. However, quantitative PET imaging of small lesions still require further investigations.

## Conclusions

PSF reconstruction artifacts can lead to misinterpretations when used for quantitative analyses of small sub-centimeter lesion, e.g., when monitoring treatment response on lymph nodes using SUV measurements. Recovery coefficients were non-monotonic as function of lesion size, less reproducible, and more slowly converging. Thus, it could be safer to use non-PSF reconstruction for quantitative purposes unless PSF reconstruction parameters are optimized to suppress PSF artifacts.

## Additional files

Additional file 1: Figure A1. The mold used to produce the twelve spheres [15]. The mold has an upper and lower part that can be split. A thin 0.4 mm diameterfishing line is placed through twelve spheres before assembling the two parts. The ^18^F-gel mixture is withdrawn into a syringe and injected into the lower part of the mold to avoid air bubbles. The vertical hole in the top and bottom of each sphere has a 0.6 mm inner diameter. Thus, the spheres can only be filled with an ^18^F mixture that is homogenous. The ^18^F mixture is held in place by capillary forces until it settles. Then, the two parts of the mold is split, and a string of 12 homogenous spheres are released. **Figure A2.** Top: A string with twelve ^18^F spheres (yellow) in air. Bottom: A string with twelve^18^F spheres (yellow) suspended in ^11^C silicone (blue). (DOCX 185 kb)
