# Supplementary material for: Point-spread function reconstructed PET images of sub-centimeter lesions are not quantitative
Source: EJNMMI Phys. 2017 Jan 13;4:5. doi: 10.1186/s40658-016-0169-9 (PMC5236043; doi:10.1186/s40658-016-0169-9)
Supplement: Additional file 1: Figure A1. — The mold used to produce the twelve spheres [15]. The mold has an upper and lower part that can be split. A thin 0.4 mm diameterfishing line is placed through twelve spheres before assembling the two parts. The 18F-gel mixture is withdrawn into a syringe and injected into the lower part of the mold to avoid air bubbles. The vertical hole in the top and bottom of each sphere has a 0.6 mm inner diameter. Thus, the spheres can only be filled with an 18F mixture that is homogenous. The 18F mixture is held in place by capillary forces until it settles. Then, the two parts of the mold is split, and a string of 12 homogenous spheres are released. Figure A2. Top: A string with twelve 18F spheres (yellow) in air. Bottom: A string with twelve18F spheres (yellow) suspended in 11C silicone (blue). (DOCX 185 kb) [file 40658_2016_169_MOESM1_ESM.docx]

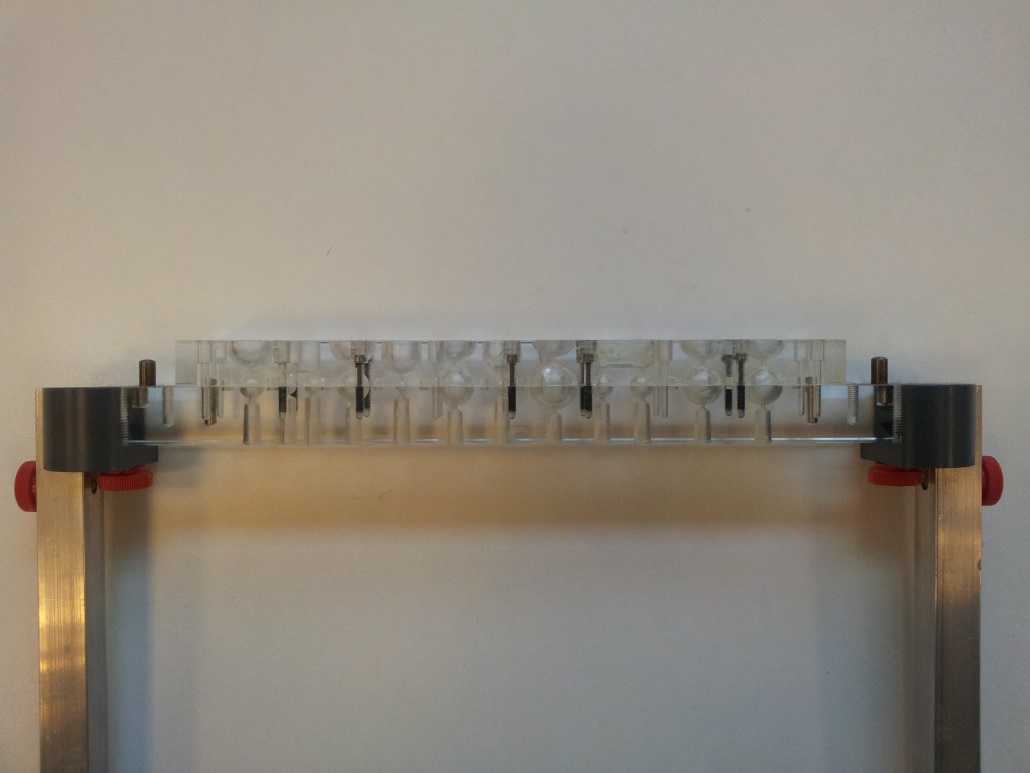
**Supplementary material**


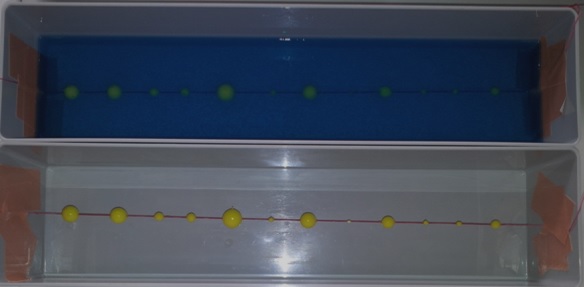

*Additional file 1: Figure A1. The mold used to produce the twelve spheres [15]. The mold has an upper and lower part that can be split. A thin 0.4 mm diameter fishing line is placed through twelve spheres before assembling the two parts. The ^18^F-gel mixture is withdrawn into a syringe and injected into the lower part of the mold to avoid air bubbles. The vertical hole in the top and bottom of each sphere has a 0.6 mm inner diameter. Thus, the spheres can only be filled with an ^18^F mixture that is homogenous. The ^18^F mixture is held in place by capillary forces until it settles. Then, the two parts of the mold is split, and a string of 12 homogenous spheres are released.*

*Additional file 1: Figure A2. Top: A string with twelve ^18^F spheres (yellow) in air. Bottom: A string with twelve ^18^F spheres (yellow) suspended in ^11^C silicone (blue).*
